# Acute Toxicity-Supported Chronic Toxicity Prediction: A *k*-Nearest Neighbor Coupled Read-Across Strategy

**DOI:** 10.3390/ijms160511659

**Published:** 2015-05-21

**Authors:** Swapnil Chavan, Ran Friedman, Ian A. Nicholls

**Affiliations:** 1 Bioorganic and Biophysical Chemistry Laboratory, Department of Chemistry and Biomedical Sciences and Linnaeus University Centre for Biomaterials Chemistry, Linnaeus University, SE-391 82 Kalmar, Sweden; 2 Computational Chemistry and Biochemistry Group, Department of Chemistry and Biomedical Sciences and Linnaeus University Centre for Biomaterials Chemistry, Linnaeus University, SE-391 82 Kalmar, Sweden; E-Mail: ran.friedman@lnu.se; 3 Department of Chemistry-BMC, Uppsala University, Box 576, SE-751 23 Uppsala, Sweden

**Keywords:** *k*-nearest neighbor, classification model, Estate fingerprint, LD_50_, LOEL, read-across, category formation

## Abstract

A *k*-nearest neighbor (*k*-NN) classification model was constructed for 118 RDT NEDO (Repeated Dose Toxicity New Energy and industrial technology Development Organization; currently known as the Hazard Evaluation Support System (HESS)) database chemicals, employing two acute toxicity (LD_50_)-based classes as a response and using a series of eight PaDEL software-derived fingerprints as predictor variables. A model developed using Estate type fingerprints correctly predicted the LD_50_ classes for 70 of 94 training set chemicals and 19 of 24 test set chemicals. An individual category was formed for each of the chemicals by extracting its corresponding *k*-analogs that were identified by *k*-NN classification. These categories were used to perform the read-across study for prediction of the chronic toxicity, *i.e.*, Lowest Observed Effect Levels (LOEL). We have successfully predicted the LOELs of 54 of 70 training set chemicals (77%) and 14 of 19 test set chemicals (74%) to within an order of magnitude from their experimental LOEL values. Given the success thus far, we conclude that if the *k*-NN model predicts LD_50_ classes correctly for a certain chemical, then the *k*-analogs of such a chemical can be successfully used for data gap filling for the LOEL. This model should support the *in silico* prediction of repeated dose toxicity.

## 1. Introduction

The multiple target effect of toxicants is a significant hurdle for pharmaceutical research and for elucidating toxicity mechanisms [1]. Off-target toxicities are a particular challenge as they are commonly not readily predicted [2]. The extent to which such a target is influenced by a toxicant is also dependent upon its effective concentration [3].

Generally, an LD_50_ experiment uses a range of toxicant doses spanning from moderate to high when administered to a set of organisms (of the same species and strain). Several mechanisms take place in such an event including off-target and non-specific effects like inflammation, mitochondrial toxicity, liver toxicity, oxidative stress, competitive inhibition of transporters and drug metabolizing enzymes, *etc.* Together, all of these effects contribute to the result (LD_50_). On the other hand, in chronic toxicity experiments, the smallest dose administered every day for the time periods of 28 days, 91 days or two years that causes any detectable effect is known as the Lowest Observable Effect Level (LOEL). In order to derive an LOEL value, many effects (such as inflammation, hypothermia, locomotor activity, *etc.*) and levels of indicators (such as liver enzymes, choline esterase, albumin/globulin ratio, *etc.*) are recorded in the test animals. As LD_50_ and LOEL can both be influenced by multiple toxicity mechanisms, we have, in this study, attempted to utilize LD_50_ values for the prediction of LOEL values.

It is established that chemicals similar in molecular structure often have similar modes of action and thus exhibit similar properties [4]. This fundamental concept has been used to predict biological effects of chemicals by clustering them on the basis of their structural similarity; such a method of prediction is known as “read-across”. Thus, clustering a group of similar chemicals with a well categorized biological profile and then using them to predict biological effects of query chemicals is a powerful approach [5,6,7,8] and such a query chemical, along with its similar chemicals, could be considered as a category. 

At present, there is no predefined basis for the acceptance or exclusion of a given chemical from a category [9]. Moreover, there are as yet no standard statistical tests for validation of such a category. To overcome these drawbacks, we used the “*k*-nearest neighbor (*k*-NN)” method to build a classification model that identified *k* neighbors for every chemical in dataset. The training and test set chemicals were considered as “queries” and their corresponding *k*-neighbors were considered as “analogs”; accordingly, a query together with its *k*-analogs was considered as a single category. The robustness of this classification model was tested using statistical validation tests. To perform the read-across study, we decided to use categories formed by *k*-NN classification models, for the following reasons: (1) the optimal validation parameters shall confirm that the categories formed through *k*-NN classification are robust enough to identify structurally similar *k*-analogs and (2) further validation of these categories shall be performed through the prediction of a class for each of the query (if such a prediction is correct).

The 3R principle, *i.e.*, to reduce, refine and replace, has been widely accepted as an ethical framework for conducting animal experiments for the purpose of research [10]. In response to this, many *in vitro* and *in silico* methods have been adopted to reduce the use of animal experiments in the last few decades. In the case of repeated dose toxicity studies, the use of *in vitro* methods has been not validated to date [11]. In repeated dose toxicity studies, an endpoint can represent a multitude of biological effects that take place through different mechanisms, occur in different organ tissues, and progress with different time frames. Accordingly, this poses a challenge for quantitative structure-activity relationship (QSAR) modelers and can explain why very few attempts have been made so far to model this endpoint [12]. In this study, we have made an attempt to construct a better model for the prediction of repeated dose toxicity.

The LOEL and LD_50_ values are typically measured in milligrams per kilograms per day, *i.e.*, milligrams of chemical per kilogram of body weight administered per day. We assumed that if lethal doses (LD_50_) are in the same range (e.g., LD_50_ of query and corresponding *k*-analogs in the range of 1 to 2000 mg/kg/day or in other similar range) or within an order of magnitude in a certain chemical category, it can be possible to predict LOEL of query using LOELs of its *k*-analogs within that category. To test this hypothesis, we decided to construct *k*-NN classification models using two classes that are based on the magnitudes of LD_50_ values and use them as response variables. We then derived *k*-analogs for the chemicals (queries) within the Repeated Dose Toxicity New Energy and industrial technology Development Organization *i.e.*, RDT NEDO database. When this classification model predicts the correct class of a query, its category can be considered qualified for the further task of read-across study. In the read-across study, LOEL values of queries from the qualified categories will be calculated by taking arithmetic means of LOELs of their respective *k*-analogs.

## 2. Results and Discussion

The RDT NEDO database documented 235 effects (refer to Table S1), 41 organ tissues were examined and 11 examination items were recorded on six strains of rats of both genders. The database was then screened for “Examination item-LOEL”, “Effect-Total effect”, “Organ tissue-Whole body”, “Organism-Rat”, “Gender-Male”, “Strain-Crj:CD(SD)” and “Route-Oral (gavage)”. 

### 2.1. k-NN Classification

A classification model is a mathematical relationship between a set of fingerprints and response variables. The *k*-nearest neighbor (*k*-NN) method is a standard and sensitive classification technique [13,14,15,16,17,18]. The *k*-NN algorithm is based on the *k*-nearest neighbors classification rule described by Hart *et al.* [19]. In this algorithm, a class of each query is predicted based on the majority class of its closest *k*-neighbors (e.g., for the category where *k* = 3, if two of the three analogs are from class 2, then predicted class for the query is class 2). The closest neighbors are identified on the basis of distance matrix. Several methods of distance calculations between queries on the basis of binary data (here, fingerprints) exist to date [20]. We have selected the “Jaccard-Tanimoto” distance method for the calculation of distance matrices [21]. 

Using the *k*-NN method, we constructed eight classification models for the respective fingerprint types. Statistical parameters of those models are given in Table 1. After examination of the Non-Error Rate (NER), sensitivity, specificity and class error, we observed that the optimal *k*-NN classification model was built from Estate fingerprints. The model consisted of 79 Estate fingerprints, was associated with NER_cv_ of 0.74 for the internal set and of 0.81 for the external test set. Selection by fivefold cross validation identified an optimal value of *k* as equal to 3.

**Table 1:** Parameters of eight *k*-NN classification models.

Entry	Fingerprint		NER	*k*	Sensitivity		Specificity	
					Class 1	Class 2	Class 1	Class 2
1	CDK	Fitting	0.69	9	0.77	0.61	0.61	0.77
		CV	0.76	9	0.84	0.68	0.68	0.84
		External	0.74	9	0.79	0.70	0.70	0.79
2	Estate	Fitting	0.75	3	0.73	0.76	0.76	0.73
		CV	0.74	3	0.77	0.71	0.71	0.77
		External	0.81	3	0.71	0.90	0.90	0.71
3	Extended CDK	Fitting	0.70	9	0.80	0.61	0.61	0.80
		CV	0.74	9	0.80	0.68	0.68	0.80
		External	0.79	9	0.79	0.80	0.80	0.79
4	Graph	Fitting	0.68	3	0.75	0.61	0.61	0.75
		CV	0.70	3	0.79	0.61	0.61	0.79
		External	0.76	3	0.71	0.80	0.80	0.71
5	Klekoth-Roth	Fitting	0.68	10	0.70	0.66	0.66	0.70
		CV	0.77	10	0.77	0.76	0.76	0.77
		External	0.72	10	0.64	0.80	0.80	0.64
6	MACCS	Fitting	0.76	7	0.79	0.74	0.74	0.79
		CV	0.73	7	0.77	0.68	0.68	0.77
		External	0.72	7	0.64	0.80	0.80	0.64
7	Pubchem	Fitting	0.77	1	0.80	0.74	0.74	0.80
		CV	0.73	1	0.77	0.68	0.68	0.77
		External	0.77	1	0.64	0.90	0.90	0.64
8	Substructure	Fitting	0.68	5	0.73	0.63	0.63	0.73
		CV	0.66	5	0.80	0.53	0.53	0.80
		External	0.71	5	0.71	0.70	0.70	0.71

The sensitivity of a model represents its ability to correctly recognize a class for a given chemical (query) while specificity characterizes an ability of a particular class to decline chemicals (queries) of all other classes. The Estate fingerprint-based *k*-NN model has demonstrated a 77% success rate in predicting toxic queries (class 1) and 71% for non-harmful queries (class 2) for the training set. Similarly, this model could predict the toxic queries (class 1) and the non-harmful queries (class 2) with specificity rates of 0.71 and 0.77, respectively. A class error of 0.26 was associated with the training set queries of both the classes (*i.e.*, class 1 and 2). For the test set, the Estate fingerprint-based *k*-NN model has demonstrated a sensitivity rate of 0.71 and 0.90, and a specificity rate of 0.90 and 0.71 for class 1 and 2 queries, respectively. The class errors associated with this model were 0.19 for the queries of both the classes *(i.e.*, class 1 and 2). This model was able to correctly classify 70 of 94 training set queries and 19 of 24 test set queries. More details are provided in Tables S3 and S4.

Thus, we have confirmed that the Estate fingerprint-based model is the most statistically robust, justifying its use in read-across studies.

### 2.2. Read-Across for LOEL Prediction

The LOEL predictions of all training set and test set queries are shown in Table 2 and Table 3, respectively, along with the LOELs of their corresponding *k*-nearest neighbors. The ratio of actual and predicted LOELs has been calculated and is referred to as *fold difference* (Fold_diff).

**Table 2:** Summary of predicted lowest observed effect levels (LOELs) of all training set queries obtained by arithmetic means of LOELs of corresponding *k*-nearest analogs (3 analogs) from Estate fingerprint based *k*-NN model.

Entry	LOEL					Fold_diff
	Query	Analog 1	Analog 2	Analog 3	Predicted	
1	30	50	625	1.2	225.40	7.51
2	50	30	625	1.2	218.73	4.37
3	200	100	750	150	333.33	1.67
4	10	10	250	30	96.67	9.67
5	30	20	30	30	26.67	1.12
6	70	300	20	20	113.33	1.62
7	5	150	200	6	118.67	23.73
8	100	200	750	200	383.33	3.83
9	150	200	100	30	110.00	1.36
10	1000	1000	11	100	370.33	2.70
11	30	1000	100	150	416.67	13.89
12	0.75	5	6	50	20.33	27.11
13	30	20	200	10	76.67	2.56
14	3130	1000	100	600	566.67	1.84
15	100	300	750	40	363.33	3.63
16	10	10	250	30	96.67	9.67
17	30	60	60	50	56.67	1.89
18	1000	30	1000	1000	676.67	1.47
19	60	30	60	50	46.67	1.29
20	600	3130	160	62.5	1117.50	1.86
21	20	30	200	10	80.00	4.00
22	750	1000	100	200	433.33	1.73
23	25	30	250	10	96.67	3.87
24	200	300	100	750	383.33	1.92
25	1000	3130	100	100	1110.00	1.11
26	250	200	3130	70	1133.33	4.53
27	200	30	20	200	83.33	2.40
28	300	200	200	100	166.67	1.80
29	160	600	1000	1000	866.67	5.42
30	350	1000	625	625	750.00	2.14
31	60	200	750	40	330.00	5.50
32	100	240	250	10	166.67	1.67
33	100	200	100	1000	433.33	4.33
34	30	30	30	100	53.33	1.78
35	3130	2500	10	350	953.33	1.09
36	1000	1000	11	30	347.00	2.88
37	40	30	60	60	50.00	1.25
38	100	40	20	30	30.00	1.11
39	1000	750	100	100	316.67	1.05
40	300	70	20	250	113.33	2.65
41	200	150	100	30	93.33	2.14
42	30	100	300	200	200.00	6.67
43	30	30	20	30	26.67	1.12
44	5	10	3130	2500	1880.00	376.00
45	40	100	300	1000	466.67	11.67
46	2	20	30	30	26.67	13.33
47	1.2	30	625	500	385.00	320.83
48	240	100	250	10	120.00	2
49	6	11	100	150	87.00	14.50
50	250	100	240	40	126.67	1.97
51	11	1000	6	1000	668.67	60.79
52	2	30	1000	30	353.33	176.67
53	62.5	6	600	3130	1245.33	19.93
54	100	1000	1000	300	766.67	7.67
55	40	200	100	150	150.00	3.75
56	10	200	100	200	166.67	16.67
57	20	70	300	1000	456.67	22.83
58	200	300	100	100	166.67	1.20
59	300	100	1000	40	380.00	1.27
60	100	1000	30	1000	676.67	6.77
61	30	30	1000	0.78	343.59	11.45
62	20	70	20	300	130.00	6.50
63	20	30	30	100	53.33	2.67
64	30	100	150	30	93.33	3.11
65	500	250	1.2	10	87.07	1.91
66	200	781	40	60	293.67	1.47
67	1000	1000	100	240	446.67	2.23
68	625	1.2	30	60	30.40	6.85
69	10	2500	3130	1000	2210.00	221.00
70	2500	10	3130	1000	1380.00	1.81
71	100	100	150	30	93.33	1.07
72	60	30	60	50	46.67	1.29
73	50	30	60	60	50.00	1.00
74	1000	350	1000	30	460.00	2.17
75	625	625	625	350	533.33	1.17
76	0.78	350	625	625	533.33	683.76
77	40	100	240	250	196.67	4.92
78	5	5	10	3130	1048.33	209.67
79	30	1.2	625	500	375.40	12.51
80	250	20	100	500	206.67	1.21
81	2	30	60	60	50.00	25.00
82	250	10	100	240	116.67	2.14
83	30	10	10	250	90.00	3.00
84	20	100	100	750	316.67	15.83
85	6	62.5	3130	70	1087.50	181.25
86	60	781	200	350	443.67	7.39
87	6	625	625	15	421.67	70.28
88	30	30	60	60	50.00	1.67
89	100	625	625	15	421.67	4.22
90	100	150	30	200	126.67	1.27
91	781	60	200	30	96.67	2.69
92	625	625	625	350	533.33	1.17
93	15	100	625	6	243.67	16.24
94	625	625	625	350	533.33	1.17

**Table 3:** Summary of predicted LOELs of all test set queries from the Estate fingerprint-based *k*-NN model.

Sr.	Query	Analog-1	LOEL Analog-2	Analog-3	Predicted	Fold_diff
1	30	30	30	20	26.67	1.13
2	30	30	1000	6	345.33	11.51
3	1.5	200	5	0.75	68.58	45.72
4	1250	750	1000	100	616.67	2.03
5	50	781	60	250	363.67	7.27
6	0.1	30	10	10	16.67	166.67
7	1000	1000	1000	11	670.33	1.49
8	20	150	200	6	118.67	5.93
9	20	5	10	5	6.67	3.00
10	100	250	100	500	283.33	2.83
11	110	1000	1000	11	670.33	6.09
12	1000	1000	100	750	616.67	1.62
13	33	30	10	10	16.67	1.98
14	30	3130	2500	200	1943.33	64.78
15	10	781	60	30	290.33	29.03
16	300	100	300	40	146.67	2.05
17	2	30	30	10	23.33	11.67
18	200	350	1000	1000	783.33	3.92
19	125	10	250	40	100.00	1.25
20	50	1000	100	30	376.67	7.53
21	100	350	625	6	327.00	3.27
22	10	6	15	10	10.33	1.03
23	150	1000	750	200	650.00	4.33
24	4	6	625	625	418.67	104.67

In the case of internal prediction, a comparison of the predicted LOELs for queries with their experimental LOELs revealed that 71 of the 94 queries from the training set have a fold difference less than a factor of 10 (refer to Table 2). A fold difference of more than 100 was observed in only seven cases. Comparison of all queries with their associated nearest three analogs suggests that most often the structural similarity, as reflected in the 79 Estate fingerprints for each query, results in a similar biological response (refer to Tables S5 and S6). Moreover, we have sorted all queries based on correct class prediction by the Estate fingerprints based *k*-NN model (refer Table S3 for predicted class information); accordingly, two types of categories were identified: (1) Qualified category (in this category, the query class was correctly predicted); and (2) Non-qualified category (in this category, the query class was wrongly predicted).

The Estate fingerprint-based model has found 70 queries in the qualified type and 24 queries in the non-qualified type of category (Table 4).

**Table 4:** Training set queries sorted (in qualified and non-qualified categories) based on its *k*-NN model-based predicted class, and further divided based upon order of magnitude difference.

Fold_diff^#^	Number of Queries		Total
	Qualified Category	Non-Qualified Category	
<10	54	17	71
10–100	12	4	16
>100	4	3	7
**Total**	70	24	94

# over of magnitude, fold differences (Fold_diff) < 10, 10–100 and >100.

The comparison of the predicted LOELs and the experimental LOELs of queries showed that 54 of the 70 queries from the qualified type of category and 17 of the 24 queries from the non-qualified type of category have less than one order of magnitude difference (fold_diff < 10). 

The LOEL values for 17 of 24 external test set queries were predicted within a factor of 10 from that of the experimental values. Only two of the 24 queries were predicted to have LOEL values that differed by more than 100-fold (Table 3).

Additionally, we have performed an analysis of categories by sorting queries into the two types of categories, *i.e.*, qualified types and non-qualified types. The comparison of the predicted and experimental LOEL of test set queries has shown that 14 of the 19 queries (74%) from the qualified type of category and 3 of the 5 queries (60%) from the non-qualified type of category have a fold difference less than 10 (Table 5).

**Table 5:** Test set query categorization (qualified and non-qualified) based on *k*-NN model-based predicted class, and further divided based upon order of magnitude difference.

Fold_diff^#^	Number of Queries		Total
	Qualified Category	Non-Qualified Category	
<10	14	3	17
10–100	4	1	5
>100	1	1	2
**Total**	19	5	24

# over of magnitude, fold differences (Fold_diff) <10, 10–100 and >100.

The 77% (54 of the 70 queries) success rate for training set queries and 74% (14 of the 19 queries) success rate for test set queries, shows that our approach is capable of finding qualified categories from the *k*-NN classification method to perform a read-across study for a LOEL prediction within an order of magnitude.

Our study revealed that the Estate fingerprint-based *k*-NN classification model performed well predicting LD_50_ classes for training and test set queries. The model has predicted correct classes of 89 of 118 queries from the training and the test sets. Moreover, our results showed that if the LD_50_ query class was predicted correctly by the classification method, then it is more likely that its LOEL would be predicted to within an order of magnitude. Our study well establishes that 68 of 89 (76%) queries (of training and test sets) from the qualified type of category were found to have their LOEL prediction with a fold difference of less than 10.

**Figure 1:**
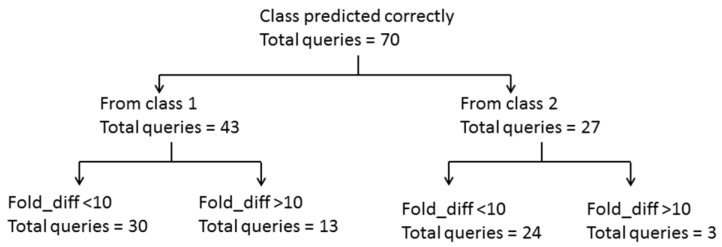
Summary of LOEL prediction for the training set queries from the qualified category.

Comparing the predictive power of this model for toxic queries (class 1) from the qualified categories revealed that 43 queries were predicted correctly (Figure 1). The LOEL prediction for 30 of 43 queries was within an order of magnitude. Of the remaining 13 queries, 10 had their LOELs predicted to within 10–100-fold of the experimental value, and the remaining 3 had >100-fold difference. Out of these 13, ten queries (*i.e.*, entries **7**, **12**, **46**, **47**, **49**, **52**, **57**, **76**, **81** and **87**) (Table 2) were extrapolated. Extrapolation is the procedure in read-across where endpoint information from category members at one end of the category is used to predict the endpoint of those members at the other end. These ten queries had the lowest LOEL in their particular categories. Thus, their predicted LOEL was calculated using members of the other side (*i.e.*, the higher LOEL side) in their respective categories, which resulted in values that were too large. The remaining three queries had their LOELs predicted between 10–20 times the experimental values: for entries **61**, **79** and **93** (Table 2) ≈ 11, 13 and 16, respectively. Among the 30 queries whose LOELs were predicted within an order of magnitude, entry **55** (in Table 2) was extrapolated, but the LOEL differences among all its analogs were less than 10-fold, and, thus, this query was predicted within an order of magnitude. 

There were 27 queries in the qualified category that belonged to class 2. Only three queries were predicted with more than a 10-fold difference. The LOEL of entry **53** (in Table 2) was predicted to within a factor of 20 from the experimental value, while the remaining two queries, entries **56** and **69** (Table 2), were extrapolated for their LOEL predictions. As their predicted LOELs were calculated using category analogs of higher LOELs, LOELs of both these entries were thus predicted with more than a 10-fold difference.

In the case of the test set, out of 19 qualified category queries, ten belonged to class 1 and nine were from class 2. Six out of ten toxic queries (class 1) and eight out of nine non-harmful (class 2) queries were predicted to within an order of magnitude. A total of five queries (four from class 1 and one from class 2) were predicted with more than 10-fold differences, three of them (*i.e.*, entry **15**, **17** and **24** (Table 3)) were extrapolated and, thus, their LOELs were predicted with more than 10-fold difference. While the remaining two queries *(i.e.*, entries **2** and **3** (Table 3)) were predicted with a fold difference of 12 and 46, respectively. Further analysis of entry **3** revealed that, in this category, analog 3 (acrolein) is its own metabolite. The entry **3** and its metabolite (acrolein) act mainly by Michael addition to exhibit their toxicity (Table 6). While, analog 2 (triallyl isocynurate) forms iminium ion that acts by S_N_1 mechanism, whereas analog 1 (1,4-butanediol) forms active metabolite gamma-hydroxy butyric acid, which is CNS depressant. As per toxic hazard classification by Crammer (with extension) [22], the class of hazard for 1,4-butanediol is low while acrolein and triallyl isocynurate have been indicated in the high toxicity class. This explains why this category fails to predict LOEL of entry **3**. Our study has correctly predicted entry **22** from test set (Table 6), where all three analogs act with similar mechanism of actions by forming reactive oxygen species [23,24]. The entry **24** was predicted wrongly as LOEL is extrapolated.

### 2.3. Mechanistic Interpretation

Our model has correctly predicted the classes of queries of specific structural scaffolds such as nitrobenzene, aniline, halogenated hydrocarbons from class 1 (toxic). The influence of substituent electronic effects is represented by the Estate fingerprints [25]. The Estate fingerprint “ddsN” represents the nitro group, “aaCH” represents aromatic carbons, fingerprint “sNH2”, “aaCH:, and “sCl” collectively represent aniline derivatives and fingerprints “ssCH2”, “sCH3”, “sF”, “sCl”, “sBr” and “sI” collectively represent halogenated hydrocarbons. The nitro aromatics and aniline derivatives are known to form reactive oxygen species (ROS) which can lead to oxidative stress and electrophilic adduct formation with tissue proteins [23]. Halogenated hydrocarbons act by S_N_2 electrophilic reaction to form adduct with DNA or proteins [26].

The correctly predicted class 2 queries are aliphatic alcohols and methacrylate esters. The Estate fingerprints “sOH”, “ssCH2”and “sCH3” collectively represent aliphatic alcohols and fingerprints “dCH2”, “dO” and “ssO” collectively represent methacrylate esters. Most of the alcohols are metabolized by the enzyme alcohol dehydrogenase to form either inactive or active metabolites. It has been shown in the literature that LD_50_ of methacrylates was related to lipophilicity [27], and they act as Michael acceptors [28].

**Table 6:** Test set query categories with their 3 respective analogs.

Entry	Data	Query	Analog 1	Analog 2	Analog 3	LOEL Predicted	Fold_diff
3	Structure	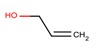	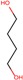	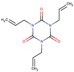	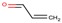		
	LD_50_	64	1525	1000	26		
	LOEL	1.5	200	5	0.75	68.58	45.72
22	Structure	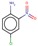	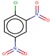	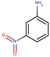	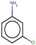		
	LD_50_	400	640	535	256		
	LOEL	10	6	15	10	10.33	1.03
24	Structure	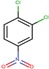	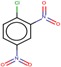	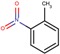	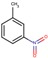		
	LD_50_	953	640	891	1072		
	LOEL	4	6	625	625	418.67	104.67

### 2.4. Comparison with Previously Published Models for Repeated Dose Toxicity Prediction

Other models for repeated dose toxicity endpoints are listed in Table 7. Comparing our study results with previous published models for LOEL endpoints, our model has shown better predictive power than studies published by De Julian-Ortiz *et al.* [29], Mazzatorta *et al.* [30] and Gadaleta *et al.* [24]. The Sakuratani *et al.* [31] study had only categorized chemicals into 33 chemical categories, while in our study we formed new categories for each of the chemicals to facilitate better prediction of their LOELs. The study performed by Mumtaz *et al.* [32] used 234 chemicals for construction of the QSAR model, but authors did not confirm the predictive power of this model using an external test set, thus it is not possible to compare our results with this model. The Garcia-Domenech *et al.* [33] study has shown slightly better predictive power than our model, but authors have used Integrated Testing Strategy (ITS), which is computationally time expensive. Our study is advantageous in comparison to other previous studies, since we have used 2D fingerprints that are fast and easy to calculate by a freely available computer program [34]. Our study has also not incorporated any difficult methods of descriptor selection that would have made this task more cumbersome and time consuming.

Furthermore, this is the novel category-approach that has taken into consideration the acute toxicity information (LD_50_ based classes) for predicting LOELs of queries in their respective categories. There are published models that have used acute toxicity data for the prediction of chronic toxicity data. Kenega [35] introduced the concept of acute/chronic ratios (ACRs). Subsequently, Rand *et al.* [36] derived ACRs by dividing the acute measure for a particular organism by its chronic measure. Kumar *et al.* [37] have developed linear regression of LogLC_50_ against inverse of exposure time (log-inverse method), the intercept of the regression was then used to estimate chronic toxicity. All these approaches only relied on biological endpoints and no theoretical information (description of chemicals) was taken into account for predicting chronic toxicity data.

**Table 7:** Literature survey of QSAR models for prediction of repeated dose toxicity endpoint.

Method	Training Set Chemicals	Test Set Chemicals	Training Set Prediction	Test Set Prediction	Comment	Reference
Multivariate analysis	234	none	95% within factor of 5	none	No external prediction	[32]
MLR	234	none	*R*^2^ = 0.52	none		[29]
MLR	86	16	*R*^2^ = 0.79	*R*^2^ = 0.71		[33]
PLS	445	none	*R*^2^ = 0.54	none	No external prediction	[30]
Read-across	500	none	none	none	33 chemical categories formed	[31]
*k*-NN	254	179	*q*^2^ = 0.63	*R*^2^ = 0.54		[24]

While in our study we have not directly used LD_50_ to predict LOELs of chemicals, we have instead formed LD_50_-based classes to identify *k*-neighbors for each chemical using *k*-NN method. Then, we have incorporated fingerprints that describe the molecular structure of chemicals. Subsequently, quantitative structural activity relationships were found among all the training set chemicals with the two classes (*i.e.*, toxic and non-harmful) by means of *k*-NN algorithms. Finally, LOELs of chemicals have been calculated by taking arithmetic mean of LOELs of their respective *k*-analogs, provided that their LD_50_ based classes have been correctly predicted. 

### 2.5. Toxicological Significance

The significance of this study is supported by the notable relationship found between different mechanisms of acute (LD_50_) and chronic toxicity (LOEL), e.g., the acute toxicity effect of liver toxicity is well explained by some of the chronic toxicity effects such as liver serum indicator and liver hypertrophy. Similarly, the mitochondrial toxicity is explained by hypothermia; the kidney toxicity is explained by creatinine, chloride, and serum protein levels as well as urine volume; the locomotor activity is explained by choline esterase level, *etc.*

It has been observed that the Estate fingerprints-based model has identified structurally similar *k*-analogs for queries. The comparison of their structures revealed that they could exhibit similar modes of actions, e.g*.*, the category for entry **22** has revealed that the query along with three analogs could possibly form reactive oxygen species, and it is very likely that they will react towards similar receptors for exhibiting their toxic actions, while in some cases, our approach has failed to derive structurally similar *k*-analogs for the query. In those categories, all members do not follow similar modes of actions (e.g*.*, entry **3**), and thus LOEL predictions can’t be performed.

## 3. Experimental Section

### 3.1. Software and Modules

The classification_toolbox Matlab module developed at the Milano Chemometrics and QSAR Research Group, University of Milan, Italy [38] was employed for the development of *k*-NN classification model. The classification_toolbox Matlab module is freely available at: http://michem.disat.unimib.it/chm/download/classificationinfo.htm.

### 3.2. Setting of the Dataset

The New Energy and Industrial Technology Development Organization (NEDO) 2007–2010 employed a database of chemicals for repeated dose toxicity endpoint in the development of the Hazard Evaluation Support System (HESS) integrated platform [39]. This database was incorporated in the OECD QSAR toolbox version 2.2 [40,41]. The 279 substances were retrieved from the RDT NEDO database using the OECD QSAR toolbox 2.2. These substances were each authenticated with respect to structure, IUPAC name and CAS registry number (RN). The SMILES notations of incorrectly assigned substances were corrected and missing SMILES notations were retrieved by using ChemSpider (http://www.chemspider.com/) [42], PubChem (http://pubchem.ncbi.nlm.nih.gov) [43] and SigmaAldrich (http://www.sigmaaldrich.com) [44]. Salts and mixtures were excluded from the dataset, as was a single chemical containing a fluorenone ring due to the lack of bulky polycyclic structures in our dataset. The resulting data set was comprised of 224 chemicals and their respective LOEL values (organism-rat, route-oral).

Acute toxicity (LD_50_) values (organism-rat, route-oral) for 134 of the 224 chemicals were found using the Toxnet (http://toxnet.nlm.nih.gov/index.html) [45] web server. Among those 134 chemicals, 16 were found to have LOEL values larger than LD_50_ values and were thus discarded from the dataset, as this implied the presence of a fundamental problem with the data underlying these 16 particular chemicals. The LOEL values for 118 chemicals were obtained by assays of varied duration (such as 28, 42, 44, 46, 49, 56, 90, 91 and 98 days, as summarized in Table S2). We included data from all assays for completeness. The selected 118 chemicals (refer to Table S2) were then classified into one of the two classes (*toxic* and *non-harmful*) using the Globally Harmonized Scheme (GHS) [46], see Table 8. These 118 chemicals were randomly divided into a training set (94 chemicals, ≈80%) and test set (24 chemicals, ≈20%) based on the principle of keeping 80% chemicals from each class in to a training and 20% chemicals from each class in to a test set.

**Table 8:** Classifications of the 118 chemicals in the training and test sets prior to *k*-NN model construction.

	Description	LD_50_ (mg/kg/day)	Number of Entries	Training Set Entries	Test Set Entries
Class 1	Highly toxic, toxic and harmful	≤2000	70	56	14
Class 2	Non-harmful	>2000	48	38	10

### 3.3. Fingerprint Calculations

Eight types of fingerprints were employed for the development of classification models. These fingerprints were calculated using the PaDEL software [34]. The PaDEL software calculates fingerprints mainly using the Chemistry Development Kit [47]. In addition, it has incorporated additional fingerprints that include atom type electro-topological state descriptors, binary fingerprints and chemical substructures count identified by Klekotha and Roth. We considered eight types of fingerprints and those are: Estate (length-79), CDK (length-1024), Extended CDK (length-1024), CDK Graph (length-1024), Pubchem (length-881), MACCS (length-166), Substructural (length-307) and Klekotha-Roth (length-4860). Each of the eight types of fingerprints was used separately to construct a classification model.

### 3.4. Development of the Classification Model

The “Jaccard-Tanimoto” distance method for calculation of distance matrices was employed for chemical classification [21]. In *k*-NN, the *k* stands for the number of neighbors to be considered. Thus, while applying *k*-NN algorithm, the optimal value of *k* needs to be determined. We have used cross validation to determine the optimal number of nearest neighbors (*k*), where a series of *k* values was assigned (from *k* = 1 to 10); based on lowest class error, an optimal *k* value was identified. The fivefold cross validation was implemented. Four groups were used for testing the class membership of the omitted group, where the class of the majority of *k* neighbors was assigned to the member of the omitted group. The *k-*NN method provided a final output for all eight types of fingerprints. All these models were later validated using the external test set.

### 3.5. External Validation

An external validation demonstrates the true predictability of a model. The test set of 24 chemicals, which were not considered for the model calibration, was used for an external validation of the model. Several validation parameters were studied to evaluate an optimum model such as non-error rate (NER), sensitivity, specificity and class error. 

### 3.6. Model Selection and Read-Across

The parameters for the internal and external validations were used in order to identify the most robust model, which was used in subsequent read-across studies. We have considered all training and test set chemicals as “queries”. By applying the *k*-NN approach, *k*-neighbors were identified for every query; each was called as its “analog”. A particular query with its corresponding *k*-analogs was considered as a single category. To predict the LOELs of each query in its category, we took the arithmetic mean of the LOELs of all the *k*-neighbors of each query.

## 4. Conclusions

A recent report from the European Chemical Agency (ECHA) has highlighted the potential of the “read-across” method to fill toxicological information data gaps [48]. At present, there is no existing rule or criteria for the acceptance or elimination of analogs from a category that is needed for read-across studies. There are also no rules for the validation of a category [9] since LD_50_ data can be used in the setting of dose levels for chronic toxicity studies [49]. Both endpoints are also influenced by multiple mechanisms including off-target and non-specific effects. Thus, we suggest a new approach for supporting the acceptance of a category for the execution of read-across, *i.e.*, if the classification model could predict correctly the class of query (toxic or non-harmful, based on LD_50_ values) by means of a *k*-NN approach, then such a correctly predicted query and its corresponding *k*-analogs can be used to perform a read-across study for the prediction of LOEL of a query.

Thus, we have successfully demonstrated the applicability of a read-across–*k*-NN coupled strategy for the prediction of repeated dose toxicity (LOEL) using acute toxicity (LD_50_) based classes. This approach should provide researchers with a tool to fill data gaps and allow the prediction of sub-chronic or chronic toxicity. This study should benefit computational toxicology, pharmacologists and risk assessors for carrying out read-across studies for the prediction of toxicological endpoints. Ultimately, this novel read-across–*k*-NN coupled strategy should contribute to a reduction in the number of animals used for chronic toxicity testing.

## Supplementary Materials

Supplementary materials can be found at http://www.mdpi.com/1422-0067/16/05/11659/s1.

## Abbreviations

CAS: Chemical Abstracts Service
CDK: Chemistry Developmental Kit
CV: Cross Validation
GHS: Globally Harmonized Scheme
IUPAC: International Union of Pure and Applied Chemistry
*k*-NN: *k* Nearest Neighbor
LOEL: Lowest Observed Effect Level
MACCS: Molecular ACCess System
NEDO: New Energy and industrial technology Development Organization
NER: Non-Error Rate
OECD: Organization for Economic Cooperation and Development
QSAR: Quantitative Structure-Activity Relationship
RDT: Repeated Dose Toxicity
SMILES: Simplified Molecular-Input Line-Entry System
