# Supplementary material for: Acute Toxicity-Supported Chronic Toxicity Prediction: A k-Nearest Neighbor Coupled Read-Across Strategy
Source: Int J Mol Sci. 2015 May 21;16(5):11659–77. doi: 10.3390/ijms160511659 (PMC4463722; doi:10.3390/ijms160511659)
Supplement: Supplementary file 1 [file ijms-16-11659-s001.pdf]

Supplementary Information

Table S1. Total 235 effects.

Sr.	Effect
1	Albumin/Globulin ↑
2	Albumin/Globulin ↓
3	Abnormal appearance
4	Abnormal behavior
5	Abnormal body position
6	Abnormal breathing
7	Abnormal fur
8	Abnormal gait
9	Abnormal response in handling
10	Abnormal sensory response
11	Absolute organ weight ↑
12	Absolute organ weight ↓
13	Alb ↑
14	Alb ↓
15	ALP ↑
16	ALP ↓
17	ALT(GPT) ↑
18	ALT(GPT) ↓
19	Amy ↑
20	Amy ↓
21	Anisonucleosis/Mitosis
22	APTT ↑
23	APTT ↓
24	AST(GOT) ↑
25	AST(GOT) ↓
26	Atrophy
27	Atrophy (necropsy)
28	Atrophy lymph follicle
29	Atrophy mucosa
30	Atrophy pelvis
31	Atrophy seminiferous
32	BASO ↑
33	BASO ↓
34	Basophilic change/Regeneration
35	Bile thrombus
36	Bilirubin ↑
37	Bilirubin ↓
38	Body weight ↑
39	Body weight ↓
40	BUN ↑
41	BUN ↓
42	Calcium ↑
43	Calcium ↓
44	Castration cell ↑

Table S1. *Cont.*

Sr.	Effect
45	Casts
46	Cell debris germ cell
47	Cell hypertrophy
48	Cell infiltration
49	Change in globulin fraction
50	ChE ↑(Plasma/RBC/Brain)
51	ChE ↓(Plasma/RBC/Brain)
52	Chloride ↑
53	Chloride ↓
54	Cl ↑
55	Cl ↓
56	Colloid ↓
57	Coma
58	Congestion
59	CPK ↑
60	CPK ↓
61	Creatinine ↑
62	CT ↑
63	CT ↓
64	Cyanosis or pale skin
65	Death
66	Degeneration nerve fiber
67	Degeneration, germ cell
68	Degeneration, semiferous tubule
69	Degeneration, vacuolar
70	Degradation epididymis
71	Degradation, nerve fiber
72	Desquamation tubular epithelium cell
73	Diarrhea/Loose stool/Bloody stool
74	Dilatation (necropsy)
75	Discoloration
76	Edema
77	emaciation
78	Enlarged
79	Enlarged (necropsy)
80	Enlarged pelvis
81	Eosinophilic body/Hyaline droplets
82	Eosinophilic hepatocyte (Centrilobular)
83	Eosinophilic hepatocyte (Other)
84	Eosinophilic hepatocyte (Periportal)
85	EOSN ↑
86	EOSN ↓
87	Erosin
88	Extramedullary hematopoiesis
89	Fatty change

Table S1. *Cont.*

Sr.	Effect
90	Fatty change pelvis
91	Fatty change/Vacuolization ↑(Other)
92	Fatty change/Vacuolization ↑(Periportal)
93	Fatty change/Vacuolization ↓(Centrilobular)
94	Fatty change/Vacuolization ↓(Other)
95	Fatty change/Vacuolization ↓(Periportal)
96	Fatty change/Vacuolization ↑(Centrilobular)
97	Fibrinogen ↑
98	Fibrinogen ↓
99	Fibrosis
100	Foamy cell accumulation
101	Food consumption ↑
102	Food consumption ↓
103	Germ cell ↓
104	Glucose ↑
105	Glucose ↓
106	Hematopoiesis extramedullary
107	Hematopoiesis ↑
108	Hematopoiesis ↓
109	Hemorrhage
110	HGB ↑
111	HGB ↓
112	HTC ↑
113	HTC ↓
114	Hyperkeratosis, Parakeratosis
115	Hyperkeratosis/Parakeratosis
116	Hyperplasia epithlium
117	Hyperplasia leydig cell
118	Hyperplasia lymph follicle
119	Hyperplasia/hypertrophy
120	Hyperplasia/Hypertrophy follicular cell
121	Hyperplasia/thickened epithelial cell
122	Hyperplasia/Thickneing epithelium
123	Hyperplasia/thicknening epithelium
124	Hyperthermia
125	Hypertrophy epithelium
126	Hypertrophy/Swelling hepatocyte (Centrilobular)
127	Hypertrophy/Swelling hepatocyte (Other)
128	Hypertrophy/Swelling hepatocyte (Periportal)
129	Hypothermia
130	I. phosphorus ↑
131	I. phosphorus ↓
132	Inflamation
133	K ↑
134	K ↓

Table S1. *Cont.*

Sr.	Effect
135	Ketone body ↑
136	Ketone body ↓
137	Lacrimation
138	LDH ↑
139	LDH ↓
140	Locomotor activity ↑
141	Locomotor activity ↓
142	LYMPH ↑
143	LYMPH ↓
144	MCH ↑
145	MCH ↓
146	MCHC ↑
147	MCHC ↓
148	MCV ↑
149	MCV ↓
150	Methemoglobin ↑
151	Mineralization
152	Miosis
153	MONO ↑
154	MONO ↓
155	Multinucleated giant cell
156	Muscle strength ↓
157	Mustle tone ↓
158	Mydriasis
159	Mydriasis
160	Myelogram
161	Myocardial degradation
162	Myocardial fibrosis
163	Myocardial necrosis
164	Na ↑
165	Na ↓
166	Necrosis
167	Necrosis germ cell
168	Necrosis hepatocyte (Centrilobular)
169	Necrosis hepatocyte (Other)
170	Necrosis hepatocyte (Periportal)
171	Necrosis pelvis
172	NEUT ↑
173	NEUT ↓
174	Occult blood
175	osmic pressure ↑
176	osmic pressure ↓
177	Other findings
178	pH ↑
179	pH ↓

Table S1. *Cont.*

Sr.	Effect
180	Pigmentation (Hemosiderin)
181	Pigmentation (Other)
182	Piloerection
183	PL ↑
184	PL ↓
185	PLT ↑
186	PLT ↓
187	Potassium ↑
188	Potassium ↓
189	Proliferation
190	Protein ↑
191	Protein ↓
192	PT ↑
193	PT ↓
194	Ptosis/Palpebral closure
195	Pupillary light reflex
196	RBC ↑
197	RBC ↓
198	Relative organ weight ↑
199	Relative organ weight ↓
200	Retention germ cell
201	Reticulocyte ↑
202	Reticulocyte ↓
203	Salivation
204	Sodium ↑
205	Sodium ↓
206	Specific gravity ↑
207	Specific gravity ↓
208	Straub tail
209	T. bilirubin ↑
210	T. bilirubin ↓
211	T. cholesterol ↑
212	T. cholesterol ↓
213	T. protein ↑
214	T. protein ↓
215	Total
216	Tremor/Convulsion
217	Triglyceride ↑
218	Triglyceride ↓
219	Ulcer
220	Urinary sediment
221	Urine volume ↑
222	Urine volume ↓
223	Urobilinogen ↑
224	Urobilinogen ↓

Table S1. *Cont.*

Sr.	Effect
225	Vacuolization
226	Vacuolization germ cell
227	Vacuolization pelvis
228	Vacuolization sertli cell
229	Vocalization
230	Water consumption ↑
231	Water consumption ↓
232	WBC ↑
233	WBC ↓
234	γ-GTP ↑
235	γ-GTP ↓

Table S2. Description of 118 chemicals and their corresponding acute and chronic toxicity values.

Sr.	CAS	LD50 (mg/kg/d)	LOEL (mg/kg/d)	SMILES	Study Period (Day)
1	100-54-9	1185	30	C(#N)c1cccnc1	28
2	100-69-6	100	50	c1(C=C)ccccn1	28
3	100-74-3	1780	200	C1CN(CC)CCO1	28
4	101-14-4	1140	10	c1(N)c(Cl)cc(Cc2cc(Cl)c(N)cc2)cc1	42
5	101-72-4	720	30	c1(NC(C)C)ccc(Nc2ccccc2)cc1	28
6	101-83-7	373	70	C1(NC2CCCCC2)CCCCC1	28
7	102-06-7	323	30	c1(NC(=N)Nc2ccccc2)ccccc1	28
8	1025-15-6	1000	5	C1(=O)N(CC=C)C(=O)N(CC=C)C(=O)N1CC=C	28
9	102-81-8	1070	100	C(CCC)N(CCCC)CCO	28
10	103-44-6	1350	30	C(CCCC)(CC)COC=C	28
11	105-16-8	4696	150	C(=O)(C(=C)C)OCCN(CC)CC	44
12	105-99-7	12,900	1000	C(=O)(CCCCC(=O)OCCCC)OCCCC	28
13	106-91-2	500	30	C(=O)(C(=C)C)OCC1CO1	45
14	107-02-8	26	0.75	C=CC=O	98
15	107-06-2	670	30	ClCCCl	91
16	1071-83-6	4873	3130	O=C(O)CNCP(=O)(O)O	91
17	107-18-6	64	1.5	C=CCO	98
18	107-66-4	3200	100	C(CCC)OP(=O)(O)OCCCC	44
19	108-42-9	256	10	Nc1cc(Cl)ccc1	93
20	108-44-1	450	30	c1(N)cc(C)ccc1	42
21	108-65-6	8532	1000	C(C)(=O)OC(C)COC	42
22	108-69-0	707	60	c1(N)cc(C)cc(C)c1	28
23	108-80-5	7700	600	C1(=O)NC(=O)NC(=O)N1	44
24	109-70-6	930	20	C(Br)CCCl	28
25	109-86-4	2370	750	OCCOC	91
26	110-02-1	1400	25	C1C=CSC=1	42
27	110-63-4	1525	200	C(O)CCCO	42
28	110-80-5	2125	1250	CCOCCO	91
29	111-17-1	3000	1000	C(=O)(O)CCSCCC(=O)O	28
30	111-41-1	3000	250	C(O)CNCCN	28
31	112-26-5	250	200	C(Cl)COCCOCCCl	28
32	115-77-5	19,500	300	C(CO)(CO)(CO)CO	46
33	118-75-2	4000	160	C1(Cl)C(=O)C(Cl)=C(Cl)C(=O)C=1Cl	28
34	119-47-1	10,000	50	C(C)(C)(C)c1c(O)c(Cc2c(O)c(C(C)(C)C)cc(C)c2)cc(C)c1	28
35	121-03-9	3710	350	c1(C)c(S(=O)(=O)O)cc(N(=O)=O)cc1	50
36	121-45-9	1600	60	COP(OC)OC	28
37	123-30-8	375	100	c1(O)ccc(N)cc1	28
38	123-42-2	2520	100	C(C)(=O)CC(C)(C)O	44
39	126-98-7	120	30	C(#N)C(=C)C	46
40	131-57-7	7400	3130	COC1=CC(=C(C=C1)C(=O)C2=CC=CC=C2)O	91
41	14047-09-7	5000	0.1	Clc2ccc(/N=N/c1ccc(Cl)c(Cl)c1)cc2Cl	91
42	141-02-6	29,200	1000	O=C(OCC(CC)CCCC)\C=C\C(=O)OCC(CCCC)CC	28
43	141-17-3	6000	1000	C(=O)(CCCCC(=O)OCCOCCOCCCC)OCCOCCOCCCC	28

Table S2. *Cont.*

Sr.	CAS	LD50 (mg/kg/d)	LOEL (mg/kg/d)	SMILES	Study Period (Day)
44	156-43-4	540	40	c1(OCC)ccc(N)cc1	28
45	2173-57-1	5930	100	c12c(cc(OCC(C)C)cc1)cccc2	28
46	2439-35-2	455	20	C(=O)(C=C)OCCN(C)C	43
47	24800-44-0	3000	1000	C(C)(O)COC(C)COC(C)CO	49
48	2579-20-6	880	300	C1(CN)CC(CN)CCC1	56
49	2867-47-2	1751	200	C(=O)(C(=C)C)OCCN(C)C	43
50	298-06-6	4510	30	C(C)OP(=S)(S)OCC	42
51	330-54-1	1017	20	c1(Cl)c(Cl)cc(NC(=O)N(C)C)cc1	42
52	40766-31-2	2050	30	c1(C)c(C)cc(C(C)c2ccccc2)cc1	28
53	4979-32-2	6420	100	c12c(cccc1)N=C(SN(C1CCCCC1)C1CCCCC1)S2	44
54	50892-23-4	1050	5	CC1=C(C(=CC=C1)NC2=CC(=NC(=N2)SCC(=O)O)Cl)C	98
55	512-56-1	840	40	COP(=O)(OC)OC	42
56	51-79-6	1809	110	CCOC(=O)N	91
57	556-61-6	72	2	C(=S)=NC	42
58	583-39-1	300	1.2	c12c(cccc1)N=C(S)N2	28
59	584-03-2	16,000	1000	C(O)(CC)CO	42
60	591-27-5	924	240	c1(O)cc(N)ccc1	28
61	591-87-7	130	6	CC(=O)OCC=C	98
62	608-93-5	1080	33	Clc1cc(Cl)c(Cl)c(Cl)c1Cl	91
63	614-45-9	1012	30	CC(C)(C)OOC(=O)C1=CC=CC=C1	91
64	620-92-8	4950	250	c1(O)ccc(Cc2ccc(O)cc2)cc1	28
65	623-91-6	1780	11	CCOC(=O)C=CC(=O)OCC	46
66	6317-18-6	55	2	N#CSCSC#N	91
67	638-16-4	9500	62.5	C1(=S)NC(=S)NC(=S)N1	42
68	6731-36-8	12,918	100	C1(OOC(C)(C)C)(OOC(C)(C)C)CC(C)(C)CC(C)C1	28
69	700-13-0	3200	10	c1(O)c(C)c(C)c(O)c(C)c1	42
70	75-50-3	500	40	CN(C)C	42
71	75-66-1	4729	10	C(C)(C)(C)S	42
72	77-73-6	353	20	C1C2C(C3C=CC2C3)CC=1	44
73	77-99-6	14,100	200	C(CC)(CO)(CO)CO	45
74	78-42-2	37,000	300	C(CCCC)(CC)COP(=O)(OCC(CCCC)CC)OCC(CCCC)CC	28
75	78-44-4	1320	100	O=C(OCC(COC(=O)NC(C)C)(C)CCC)N	91
76	78-51-3	3000	300	C(CCC)OCCOP(=O)(OCCOCCCC)OCCOCCCC	28
77	78-67-1	100	2	C(#N)C(C)(C)N=NC(C)(C)C#N	42
78	78-97-7	87	30	C(#N)C(C)O	43
79	79-27-6	1200	20	C(Br)(Br)C(Br)Br	28
80	793-24-8	3580	20	c1(NC(C)CC(C)C)ccc(Nc2ccccc2)cc1	28
81	79-39-0	459	30	C(N)(=O)C(=C)C	28
82	8003-22-3	5000	500	O=C4c1ccccc1C(=O)C4c2nc3ccccc3cc2	91
83	80-09-1	4556	200	c1(S(=O)(=O)c2ccc(O)cc2)ccc(O)cc1	28
84	80-43-3	4100	200	C(C)(C)(c1ccccc1)OOC(C)(C)c1ccccc1	28
85	81-16-3	19,400	1000	c1(N)c(S(=O)(=O)O)c2c(cccc2)cc1	49
86	822-36-6	751	625	CC1=CN=CN1	98

Table S2. *Cont.*

Sr.	CAS	LD50 (mg/kg/d)	LOEL (mg/kg/d)	SMILES	Study Period (Day)
87	84-51-5	2795	10	c12C(=O)c3c(C(=O)c1cc(CC)cc2)cccc3	28
88	84-74-2	7499	2500	CCCCOC(=O)c1ccccc1C(=O)OCCCC	91
89	86-87-3	1000	125	c1(CC(=O)O)c2c(cccc2)ccc1	28
90	868-77-9	5050	100	C(=O)(C(=C)C)OCCO	49
91	87-59-2	933	60	c1(N)c(C)c(C)ccc1	28
92	87-62-7	840	50	c1(C)c(N)c(C)ccc1	42
93	88-09-5	2200	50	C(=O)(O)C(CC)CC	42
94	88-44-8	11,700	1000	c1(N)c(S(=O)(=O)O)cc(C)cc1	28
95	88-72-2	891	625	CC1=CC=CC=C1N(=O)=O	91
96	88-85-7	25	0.78	c1(N(=O)=O)c(O)c(C(C)CC)cc(N(=O)=O)c1	42
97	88-89-1	200	100	OC1=C(C=C(C=C1N(=O)=O)N(=O)=O)N(=O)=O	28
98	89-63-4	400	10	c1(N)c(N(=O)=O)cc(Cl)cc1	42
99	92-88-6	4920	40	c1(-c2ccc(O)cc2)ccc(O)cc1	42
100	941-69-5	58	5	C1(=O)C=CC(=O)N1c1ccccc1	49
101	95-14-7	560	30	c12c(cccc1)N=NN2	42
102	95-33-0	5300	250	c12c(cccc1)N=C(SNC1CCCCC1)S2	28
103	95-68-1	467	2	c1(N)c(C)cc(C)cc1	28
104	95-85-2	690	250	c1(O)c(N)cc(Cl)cc1	42
105	95-94-3	1500	30	c1c(c(cc(c1Cl)Cl)Cl)Cl	91
106	96-29-7	930	20	C(C)(CC)=NO	28
107	96-45-7	1832	6	C1(=S)NCCN1	28
108	96-69-5	2345	60	C(C)(C)(C)c1c(O)cc(C)c(Sc2c(C)cc(O)c(C(C)(C)C)c2)c1	28
109	97-00-7	640	6	c1(Cl)c(N(=O)=O)cc(N(=O)=O)cc1	42
110	97-39-2	500	30	CC1=CC=CC=C1NC(=NC2=CC=CC=C2C)N	28
111	97-52-9	997	100	COc1cc(ccc1N)N(=O)=O	28
112	97-88-1	16,000	100	C(=O)(C(=C)C)OCCCC	44
113	97-99-4	1600	150	C1(CO)CCCO1	28
114	98-29-3	2820	781	CC(C)(C)C1=CC(=C(C=C1)O)O	98
115	99-08-1	1072	625	CC1=CC(=CC=C1)N(=O)=O	91
116	99-09-2	535	15	NC1=CC=CC(=C1)N(=O)=O	28
117	99-54-7	953	4	ClC1=C(Cl)C=C(C=C1)N(=O)=O	42
118	99-99-0	1960	625	Cc1ccc(cc1)N(=O)=O	91

Table S3. Training set Actual class *vs.* Predicted class.

Sr.	Predicted Class	Actual Class
1	1	1
2	1	1
3	2	1
4	1	1
5	2	1
6	1	1
7	1	1
8	2	1
9	1	2
10	2	2
11	2	1
12	1	1
13	1	1
14	2	2
15	2	2
16	1	1
17	1	1
18	2	2
19	1	1
20	2	2
21	1	1
22	2	2
23	1	1
24	2	1
25	2	2
26	2	2
27	1	1
28	2	2
29	2	2
30	1	2
31	1	1
32	1	1
33	2	2
34	1	1
35	2	2
36	2	2
37	1	1
38	1	2
39	2	2
40	1	1
41	2	1
42	2	2
43	1	2
44	2	1
45	2	1

Table S3. *Cont.*

Sr.	Predicted Class	Actual Class
46	1	1
47	1	1
48	1	1
49	1	1
50	1	2
51	2	1
52	1	1
53	2	2
54	1	2
55	1	1
56	2	2
57	1	1
58	2	2
59	2	2
60	2	1
61	1	1
62	1	1
63	1	2
64	1	1
65	1	2
66	1	2
67	2	2
68	1	1
69	2	2
70	2	2
71	2	2
72	1	1
73	1	1
74	2	2
75	1	1
76	1	1
77	2	2
78	2	1
79	1	1
80	2	2
81	1	1
82	1	1
83	1	1
84	2	1
85	2	1
86	2	2
87	1	1
88	1	1
89	1	1
90	1	2

Table S3. *Cont.*

Sr.	Predicted Class	Actual Class
91	1	2
92	1	1
93	1	1
94	1	1

Table S4. Test set Acual class *vs.* Predicted class.

Sr.	Predicted Class	Actual Class
1	1	1
2	1	1
3	1	1
4	2	2
5	2	2
6	1	2
7	2	2
8	1	1
9	1	1
10	2	2
11	2	1
12	2	2
13	1	1
14	2	1
15	2	2
16	2	2
17	1	1
18	2	2
19	2	1
20	2	2
21	1	1
22	1	1
23	2	1
24	1	1

Table S5. Prediction of training set compounds using 3 analogs obtained from *k*-NN classification.

Sr	Data	Target	Analog 1	Analog 2	Analog 3	LOEL Predicted	Fold_diff
1	Structure	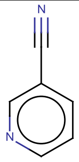	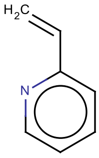	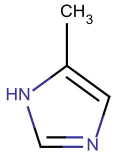	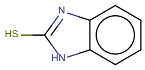		
	LD50	1185	100	751	300		
	LOEL	30	50	625	1.2	225.40	7.51
2	Structure	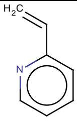	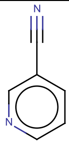	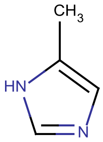	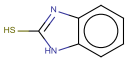		
	LD50	100	1185	751	300		
	LOEL	50	30	625	1.2	218.73	4.37
3	Structure	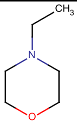	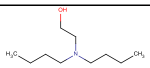	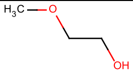	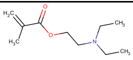		
	LD50	1780	1070	2370	4696		
	LOEL	200	100	750	150	333.33	1.67
4	Structure	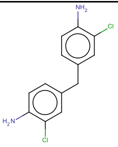	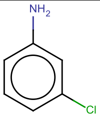	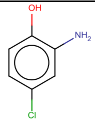	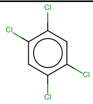		
	LD50	1140	256	690	1500		
	LOEL	10	10	250	30	96.67	9.67
5	Structure	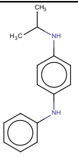	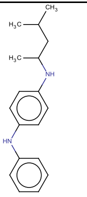	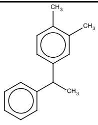	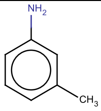		
	LD50	720	3580	2050	450		
	LOEL	30	20	30	30	26.67	1.12
6	Structure	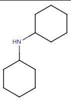	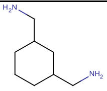	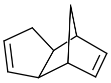	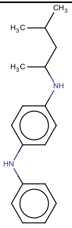		
	LD50	373	880	353	3580		
	LOEL	70	300	20	20	113.33	1.62
7	Structure	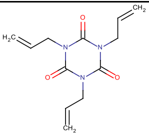	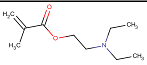	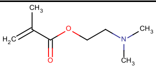	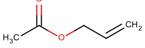		
	LD50	1000	4696	1751	130		
	LOEL	5	150	200	6	118.67	23.73

Table S5. *Cont.*

Sr	Data	Target	Analog 1	Analog 2	Analog 3	LOEL Predicted	Fold_diff
8	Structure	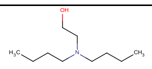	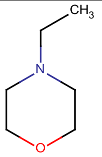	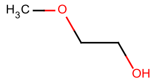	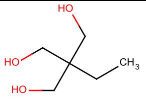		
	LD50	1070	1780	2370	14,100		
	LOEL	100	200	750	200	383.33	3.83
9	Structure	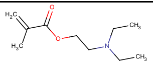	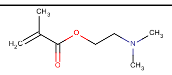	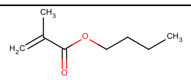	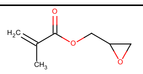		
	LD50	4696	1751	16,000	500		
	LOEL	150	200	100	30	110.00	1.36
10	Structure	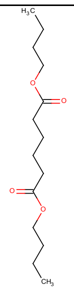	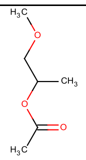	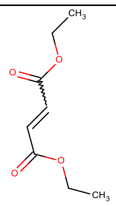	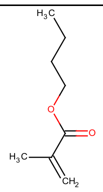		
	LD50	12,900	8532	1780	16,000		
	LOEL	1000	1000	11	100	370.33	2.70
11	Structure	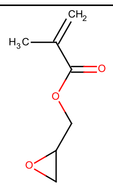	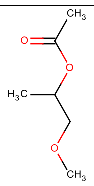	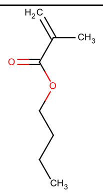	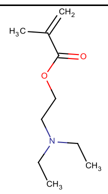		
	LD50	500	8532	16,000	4696		
	LOEL	30	1000	100	150	416.67	13.89
12	Structure	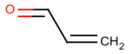	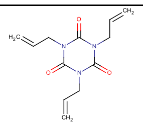	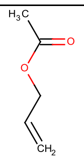	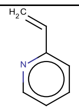		
	LD50	26	1000	130	100		
	LOEL	0.75	5	6	50	20.33	27.11
13	Structure	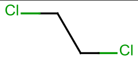	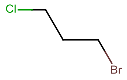	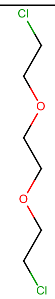	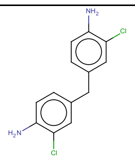		
	LD50	670	930	250	1140		
	LOEL	30	20	200	10	76.67	2.56

Table S5. *Cont.*

Sr	Data	Target	Analog 1	Analog 2	Analog 3	LOEL Predicted	Fold_diff
14	Structure	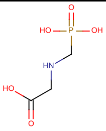	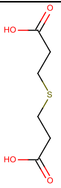	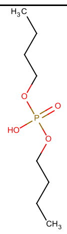	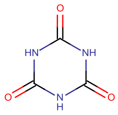		
	LD50	4873	3000	3200	7700		
	LOEL	3130	1000	100	600	566.67	1.84
15	Structure	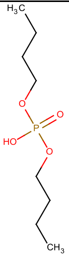	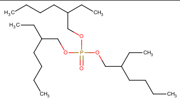	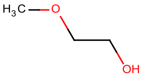	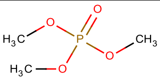		
	LD50	3200	37,000	2370	840		
	LOEL	100	300	750	40	363.33	3.63
16	Structure	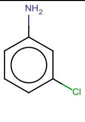	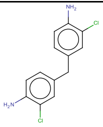	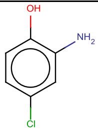	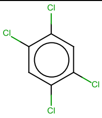		
	LD50	256	1140	690	1500		
	LOEL	10	10	250	30	96.67	9.67
17	Structure	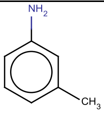	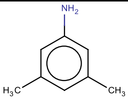	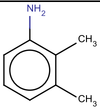	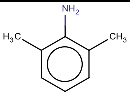		
	LD50	450	707	933	840		
	LOEL	30	60	60	50	56.67	1.89
18	Structure	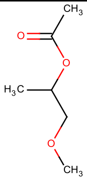	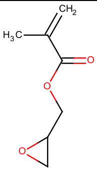	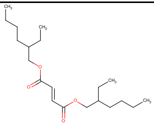	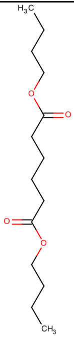		
	LD50	8532	500	29,200	12,900		
	LOEL	1000	30	1000	1000	676.67	1.47
19	Structure	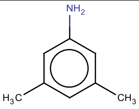	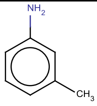	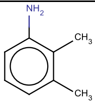	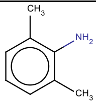		
	LD50	707	450	933	840		
	LOEL	60	30	60	50	46.67	1.29

Table S5. *Cont.*

Sr	Data	Target	Analog 1	Analog 2	Analog 3	LOEL Predicted	Fold_diff
20	Structure	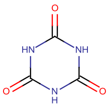	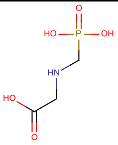	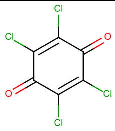	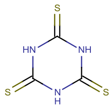		
	LD50	7700	4873	4000	9500		
	LOEL	600	3130	160	62.5	1 117.50	1.86
21	Structure	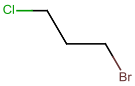	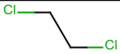	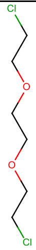	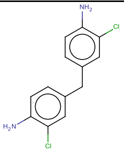		
	LD50	930	670	250	1140		
	LOEL	20	30	200	10	80.00	4.00
22	Structure	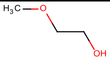	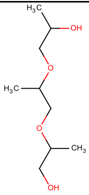	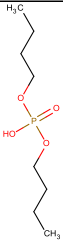	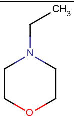		
	LD50	2370	3000	3200	1780		
	LOEL	750	1000	100	200	433.33	1.73
23	Structure	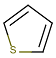	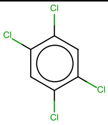	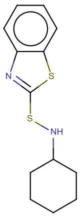	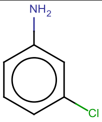		
	LD50	1400	1500	5300	256		
	LOEL	25	30	250	10	96.67	3.87
24	Structure	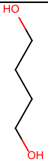	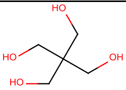	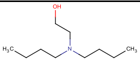	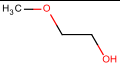		
	LD50	1525	19,500	1070	2370		
	LOEL	200	300	100	750	383.33	1.92
25	Structure	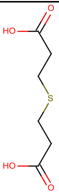	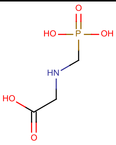	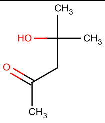	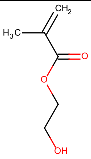		
	LD50	3000	4873	2520	5050		
	LOEL	1000	3130	100	100	1110.00	1.11

Table S5. *Cont.*

Sr	Data	Target	Analog 1	Analog 2	Analog 3	LOEL Predicted	Fold_diff
26	Structure	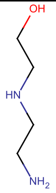	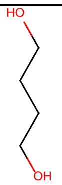	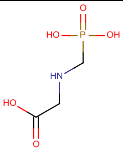	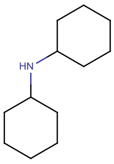		
	LD50	3000	1525	4873	373		
	LOEL	250	200	3130	70	1133.33	4.53
27	Structure	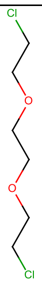	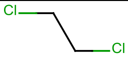	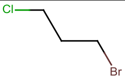	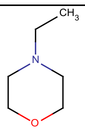		
	LD50	250	670	930	1780		
	LOEL	200	30	20	200	83.33	2.40
28	Structure	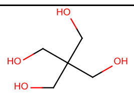	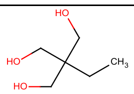	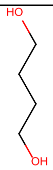	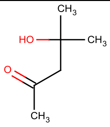		
	LD50	19,500	14,100	1525	2520		
	LOEL	300	200	200	100	166.67	1.80
29	Structure	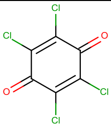	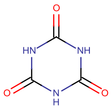	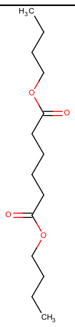	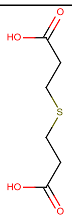		
	LD50	4000	7700	12,900	3000		
	LOEL	160	600	1000	1000	866.67	5.42
30	Structure	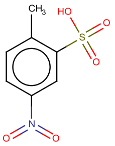	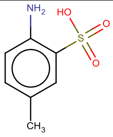	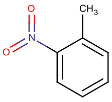	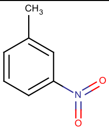		
	LD50	3710	11,700	891	1072		
	LOEL	350	1000	625	625	750.00	2.14
31	Structure	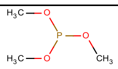	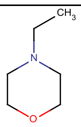	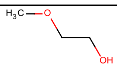	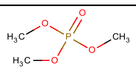		
	LD50	1600	1780	2370	840		
	LOEL	60	200	750	40	330.00	5.50

Table S5. *Cont.*

Sr	Data	Target	Analog 1	Analog 2	Analog 3	LOEL Predicted	Fold_diff
32	Structure	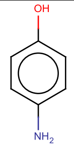	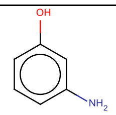	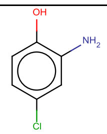	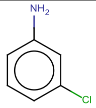		
	LD50	375	924	690	256		
	LOEL	100	240	250	10	166.67	1.67
33	Structure	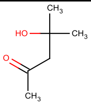	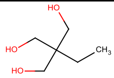	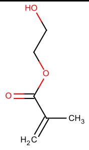	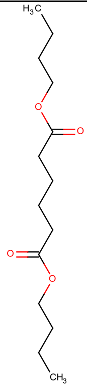		
	LD50	2520	14,100	5050	12,900		
	LOEL	100	200	100	1000	433.33	4.33
34	Structure	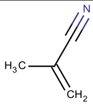	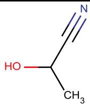	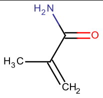	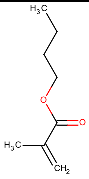		
	LD50	120	87	459	16,000		
	LOEL	30	30	30	100	53.33	1.78
35	Structure	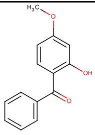	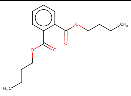	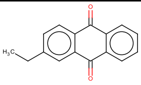	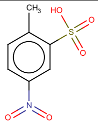		
	LD50	7400	7499	2795	3710		
	LOEL	3130	2500	10	350	953.33	1.09
36	Structure	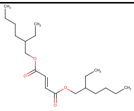	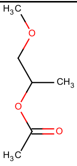	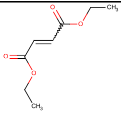	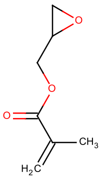		
	LD50	29,200	8532	1780	500		
	LOEL	1000	1000	11	30	347.00	2.88
37	Structure	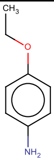	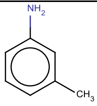	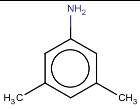	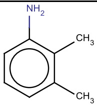		
	LD50	540	450	707	933		
	LOEL	40	30	60	60	50.00	1.25

Table S5. *Cont.*

Sr	Data	Target	Analog 1	Analog 2	Analog 3	LOEL Predicted	Fold_diff
38	Structure	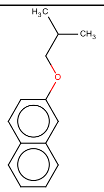	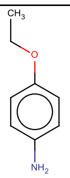	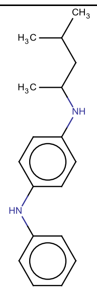	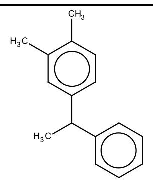		
	LD50	5930	540	3580	2050		
	LOEL	100	40	20	30	30.00	1.11
39	Structure	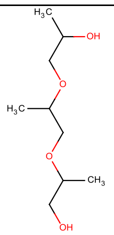	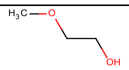	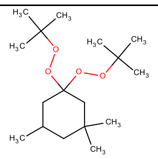	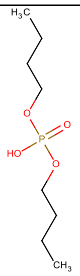		
	LD50	3000	2370	12,918	3200		
	LOEL	1000	750	100	100	316.67	1.05
40	Structure	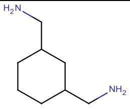	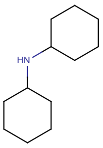	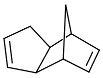	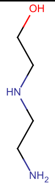		
	LD50	880	373	353	3000		
	LOEL	300	70	20	250	113.33	2.65
41	Structure	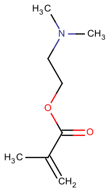	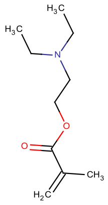	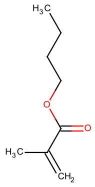	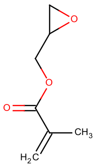		
	LD50	1751	4696	16,000	500		
	LOEL	200	150	100	30	93.33	2.14
42	Structure	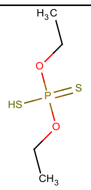	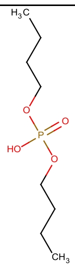	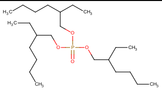	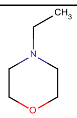		
	LD50	4510	3200	37,000	1780		
	LOEL	30	100	300	200	200.00	6.67

Table S5. *Cont.*

Sr	Data	Target	Analog 1	Analog 2	Analog 3	LOEL Predicted	Fold_diff
43	Structure	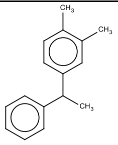	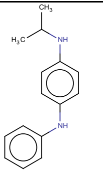	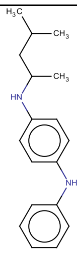	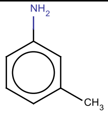		
	LD50	2050	720	3580	450		
	LOEL	30	30	20	30	26.67	1.12
44	Structure	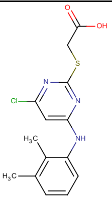	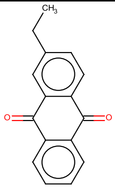	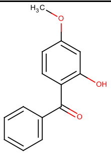	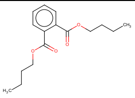		
	LD50	1050	2795	7400	7499		
	LOEL	5	10	3130	2500	1880.00	376.00
45	Structure	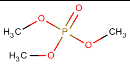	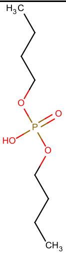	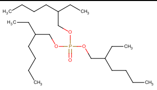	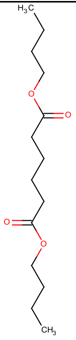		
	LD50	840	3200	37,000	12,900		
	LOEL	40	100	300	1000	466.67	11.67
46	Structure	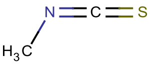	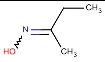	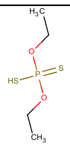	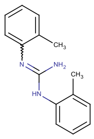		
	LD50	72	930	4510	500		
	LOEL	2	20	30	30	26.67	13.33
47	Structure	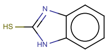	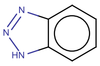	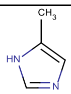	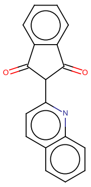		
	LD50	300	560	751	5000		
	LOEL	1.2	30	625	500	385.00	320.83
48	Structure	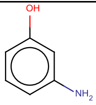	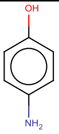	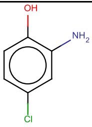	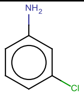		
	LD50	924	375	690	256		
	LOEL	240	100	250	10	120.00	2

Table S5. *Cont.*

Sr	Data	Target	Analog 1	Analog 2	Analog 3	LOEL Predicted	Fold_diff
49	Structure	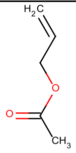	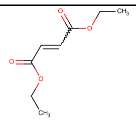	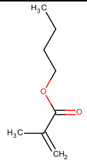	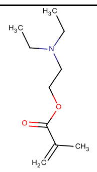		
	LD50	130	1780	16,000	4696		
	LOEL	6	11	100	150	87.00	14.50
50	Structure	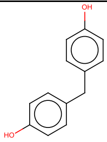	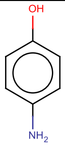	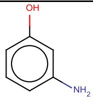	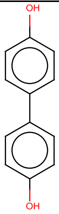		
	LD50	4950	375	924	4920		
	LOEL	250	100	240	40	126.67	1.97
51	Structure	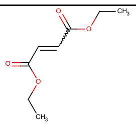	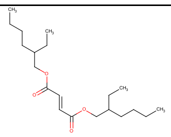	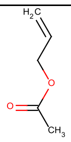	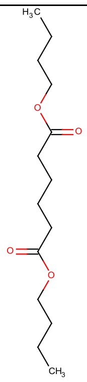		
	LD50	1780	29,200	130	12,900		
	LOEL	11	1000	6	1000	668.67	60.79
52	Structure	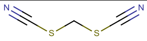	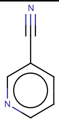	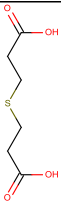	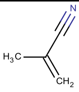		
	LD50	55	1185	3000	120		
	LOEL	2	30	1000	30	353.33	176.67
53	Structure	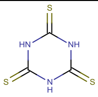	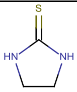	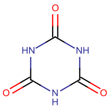	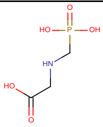		
	LD50	9500	1832	7700	4873		
	LOEL	62.5	6	600	3130	1 245.33	19.93
54	Structure	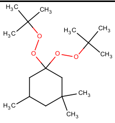	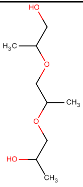	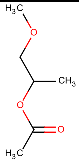	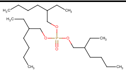		
	LD50	12,918	3000	8532	37,000		
	LOEL	100	1000	1000	300	766.67	7.67

Table S5. *Cont.*

Sr	Data	Target	Analog 1	Analog 2	Analog 3	LOEL Predicted	Fold_diff
55	Structure	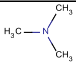	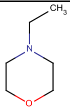	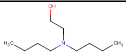	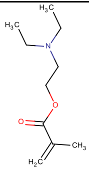		
	LD50	500	1780	1070	4696		
	LOEL	40	200	100	150	150.00	3.75
56	Structure	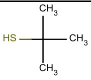	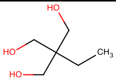	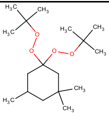	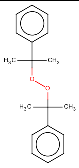		
	LD50	4729	14,100	12,918	4100		
	LOEL	10	200	100	200	166.67	16.67
57	Structure	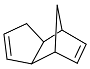	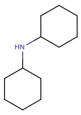	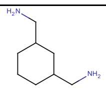	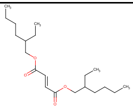		
	LD50	353	373	880	29,200		
	LOEL	20	70	300	1000	456.67	22.83
58	Structure	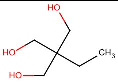	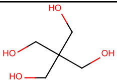	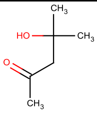	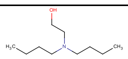		
	LD50	14,100	19,500	2520	1070		
	LOEL	200	300	100	100	166.67	1.20
59	Structure	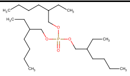	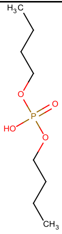	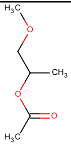	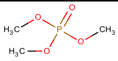		
	LD50	37,000	3200	8532	840		
	LOEL	300	100	1000	40	380.00	1.27
60	Structure	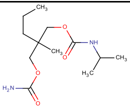	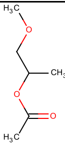	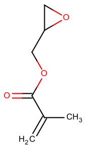	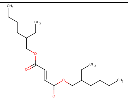		
	LD50	1320	8532	500	29,200		
	LOEL	100	1000	30	1000	676.67	6.77
61	Structure	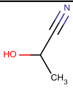	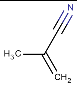	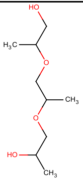	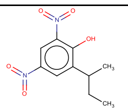		
	LD50	87	120	3000	25		
	LOEL	30	30	1000	0.78	343.59	11.45

Table S5. *Cont.*

Sr	Data	Target	Analog 1	Analog 2	Analog 3	LOEL Predicted	Fold_diff
62	Structure	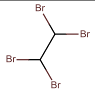	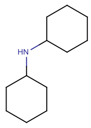	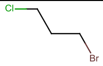	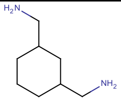		
	LD50	1200	373	930	880		
	LOEL	20	70	20	300	130.00	6.50
63	Structure	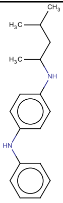	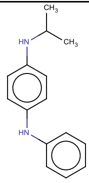	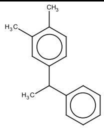	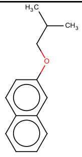		
	LD50	3580	720	2050	5930		
	LOEL	20	30	30	100	53.33	2.67
64	Structure	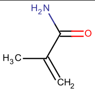	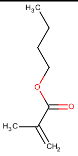	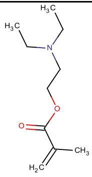	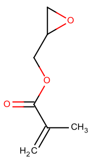		
	LD50	459	16,000	4696	500		
	LOEL	30	100	150	30	93.33	3.11
65	Structure	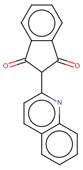	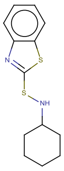	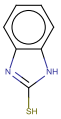	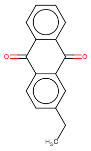		
	LD50	5000	5300	300	2795		
	LOEL	500	250	1.2	10	87.07	1.91
66	Structure	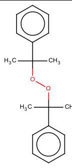	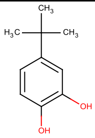	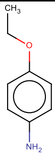	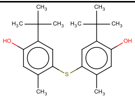		
	LD50	4100	2820	540	2345		
	LOEL	200	781	40	60	293.67	1.47
67	Structure	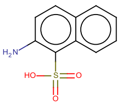	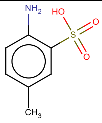	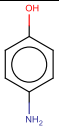	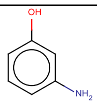		
	LD50	19,400	11,700	375	924		
	LOEL	1000	1000	100	240	446.67	2.23
68	Structure	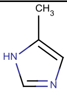	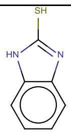	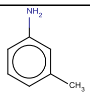	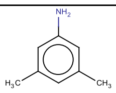		
	LD50	751	300	450	707		
	LOEL	625	1.2	30	60	30.40	6.85

Table S5. *Cont.*

Sr	Data	Target	Analog 1	Analog 2	Analog 3	LOEL Predicted	Fold_diff
69	Structure	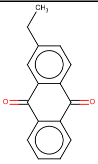	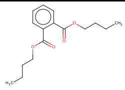	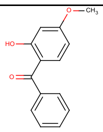	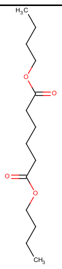		
	LD50	2795	7499	7400	12,900		
	LOEL	10	2500	3130	1000	2210.00	221.00
70	Structure	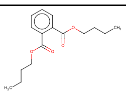	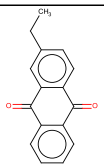	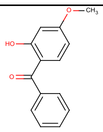	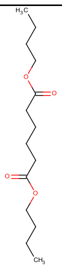		
	LD50	7499	2795	7400	12,900		
	LOEL	2500	10	3130	1000	1380.00	1.81
71	Structure	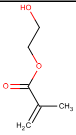	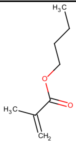	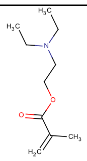	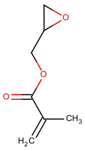		
	LD50	5050	16,000	4696	500		
	LOEL	100	100	150	30	93.33	1.07
72	Structure	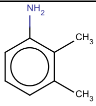	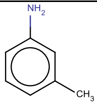	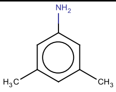	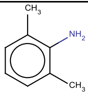		
	LD50	933	450	707	840		
	LOEL	60	30	60	50	46.67	1.29
73	Structure	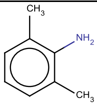	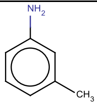	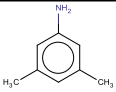	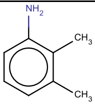		
	LD50	840	450	707	933		
	LOEL	50	30	60	60	50.00	1.00
74	Structure	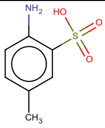	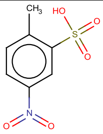	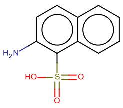	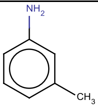		
	LD50	11,700	3710	19,400	450		
	LOEL	1000	350	1000	30	460.00	2.17
75	Structure	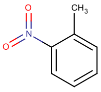	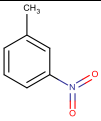	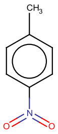	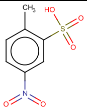		
	LD50	891	1072	1960	3710		
	LOEL	625	625	625	350	533.33	1.17

Table S5. *Cont.*

Sr	Data	Target	Analog 1	Analog 2	Analog 3	LOEL Predicted	Fold_diff
76	Structure	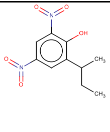	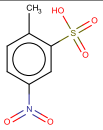	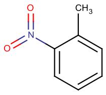	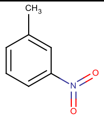		
	LD50	25	3710	891	1072		
	LOEL	0.78	350	625	625	533.33	683.76
77	Structure	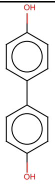	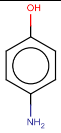	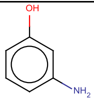	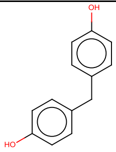		
	LD50	4920	375	924	4950		
	LOEL	40	100	240	250	196.67	4.92
78	Structure	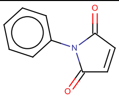	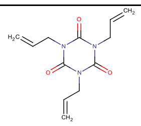	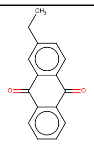	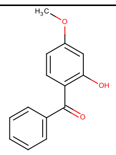		
	LD50	58	1000	2795	7400		
	LOEL	5	5	10	3130	1 048.33	209.67
79	Structure	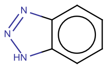	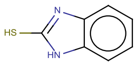	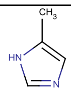	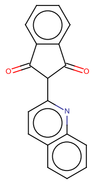		
	LD50	560	300	751	5000		
	LOEL	30	1.2	625	500	375.40	12.51
80	Structure	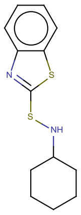	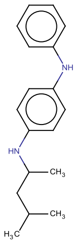	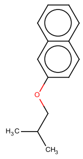	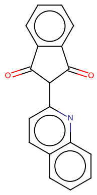		
	LD50	5300	3580	5930	5000		
	LOEL	250	20	100	500	206.67	1.21
81	Structure	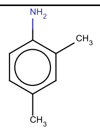	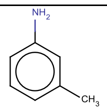	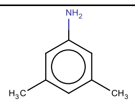	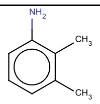		
	LD50	467	450	707	933		
	LOEL	2	30	60	60	50.00	25.00
82	Structure	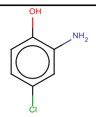	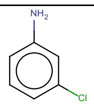	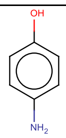	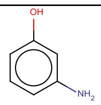		
	LD50	690	256	375	924		
	LOEL	250	10	100	240	116.67	2.14

Table S5. *Cont.*

Sr	Data	Target	Analog 1	Analog 2	Analog 3	LOEL Predicted	Fold_diff
83	Structure	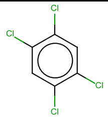	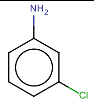	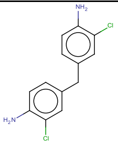	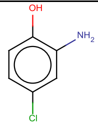		
	LD50	1500	256	1140	690		
	LOEL	30	10	10	250	90.00	3.00
84	Structure	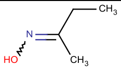	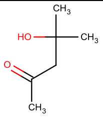	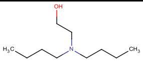	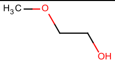		
	LD50	930	2520	1070	2370		
	LOEL	20	100	100	750	316.67	15.83
85	Structure	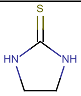	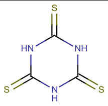	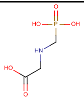	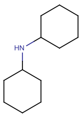		
	LD50	1832	9500	4873	373		
	LOEL	6	62.5	3130	70	1087.50	181.25
86	Structure	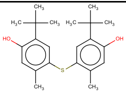	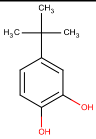	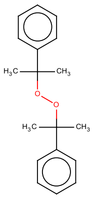	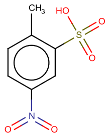		
	LD50	2345	2820	4100	3710		
	LOEL	60	781	200	350	443.67	7.39
87	Structure	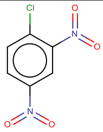	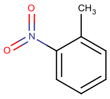	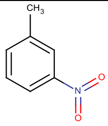	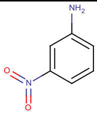		
	LD50	640	891	1072	535		
	LOEL	6	625	625	15	421.67	70.28
88	Structure	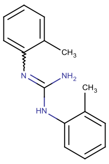	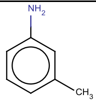	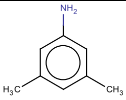	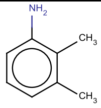		
	LD50	500	450	707	933		
	LOEL	30	30	60	60	50.00	1.67
89	Structure	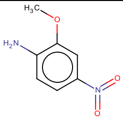	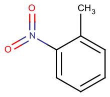	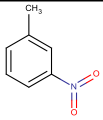	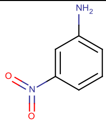		
	LD50	997	891	1072	535		
	LOEL	100	625	625	15	421.67	4.22

Table S5. *Cont.*

Sr	Data	Target	Analog 1	Analog 2	Analog 3	LOEL Predicted	Fold_diff
90	Structure	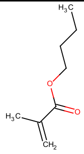	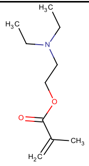	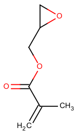	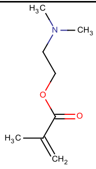		
	LD50	16,000	4696	500	1751		
	LOEL	100	150	30	200	126.67	1.27
91	Structure	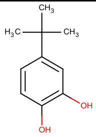	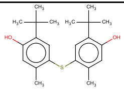	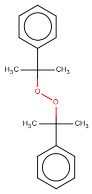	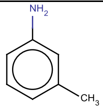		
	LD50	2820	2345	4100	450		
	LOEL	781	60	200	30	96.67	2.69
92	Structure	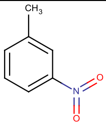	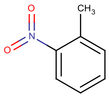	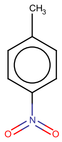	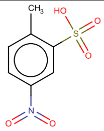		
	LD50	1072	891	1960	3710		
	LOEL	625	625	625	350	533.33	1.17
93	Structure	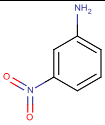	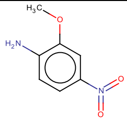	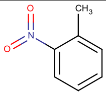	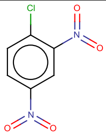		
	LD50	535	997	891	640		
	LOEL	15	100	625	6	243.67	16.24
94	Structure	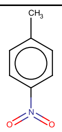	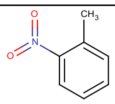	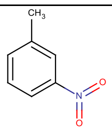	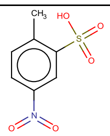		
	LD50	1960	891	1072	3710		
	LOEL	625	625	625	350	533.33	1.17

Table S6. Prediction of test set compounds using 3 analogs obtained from *k*-NN classification.

Sr	Data	Target	Ana-1	Ana-2	Ana-3	LOEL_pred	Fold_diff
1	Structure	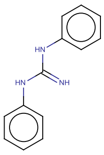	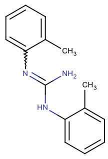	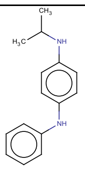	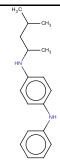		
	LD50	323	500	720	3580		
	LOEL	30	30	30	20	26.67	1.13
2	Structure	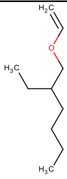	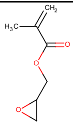	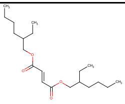	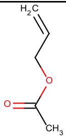		
	LD50	1350	500	29,200	130		
	LOEL	30	30	1000	6	345.33	11.51
3	Structure	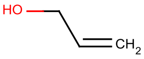	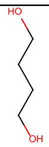	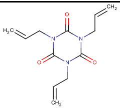	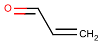		
	LD50	64	1525	1000	26		
	LOEL	1.5	200	5	0.75	68.58	45.72
4	Structure	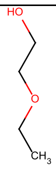	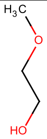	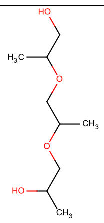	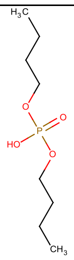		
	LD50	2125	2370	3000	3200		
	LOEL	1250	750	1000	100	616.67	2.03
5	Structure	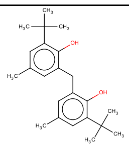	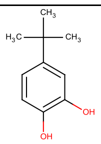	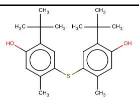	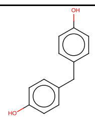		
	LD50	10,000	2820	2345	4950		
	LOEL	50	781	60	250	363.67	7.27
6	Structure	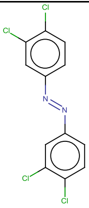	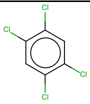	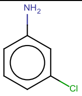	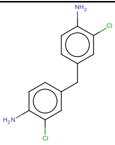		
	LD50	5000	1500	256	1140		
	LOEL	0.1	30	10	10	16.67	166.67

Table S6. *Cont.*

Sr	Data	Target	Ana-1	Ana-2	Ana-3	LOEL_pred	Fold_diff
7	Structure	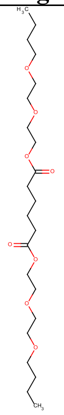	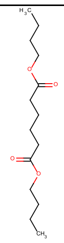	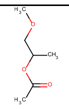	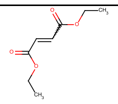		
	LD50	6000	12,900	8532	1780		
	LOEL	1000	1000	1000	11	670.33	1.49
8	Structure	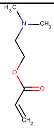	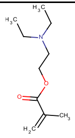	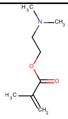	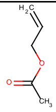		
	LD50	455	4696	1751	130		
	LOEL	20	150	200	6	118.67	5.93
9	Structure	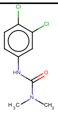	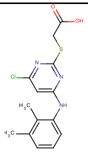	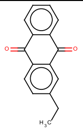	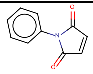		
	LD50	1017	1050	2795	58		
	LOEL	20	5	10	5	6.67	3.00
10	Structure	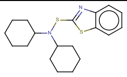	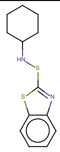	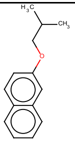	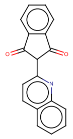		
	LD50	6420	5300	5930	5000		
	LOEL	100	250	100	500	283.33	2.83
11	Structure	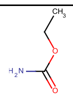	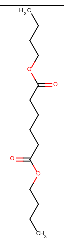	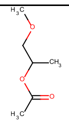	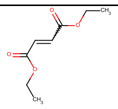		
	LD50	1809	12,900	8532	1780		
	LOEL	110	1000	1000	11	670.33	6.09
12	Structure	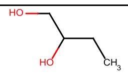	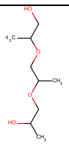	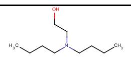	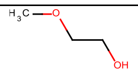		
	LD50	16,000	3000	1070	2370		
	LOEL	1000	1000	100	750	616.67	1.62

Table S6. *Cont.*

Sr	Data	Target	Ana-1	Ana-2	Ana-3	LOEL_pred	Fold_diff
13	Structure	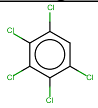	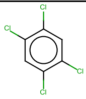	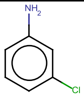	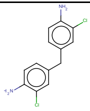		
	LD50	1080	1500	256	1140		
	LOEL	33	30	10	10	16.67	1.98
14	Structure	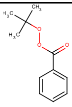	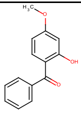	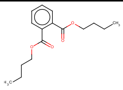	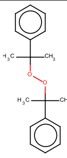		
	LD50	1012	7400	7499	4100		
	LOEL	30	3130	2500	200	1943.33	64.78
15	Structure	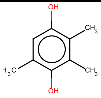	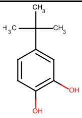	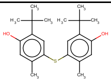	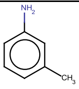		
	LD50	3200	2820	2345	450		
	LOEL	10	781	60	30	290.33	29.03
16	Structure	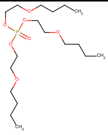	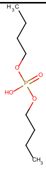	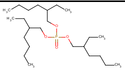	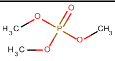		
	LD50	3000	3200	37,000	840		
	LOEL	300	100	300	40	146.67	2.05
17	Structure	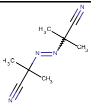	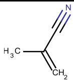	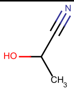	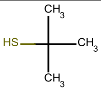		
	LD50	100	120	87	4729		
	LOEL	2	30	30	10	23.33	11.67
18	Structure	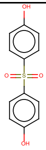	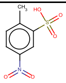	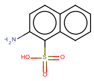	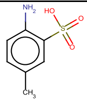		
	LD50	4556	3710	19,400	11,700		
	LOEL	200	350	1000	1000	783.33	3.92
19	Structure	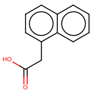	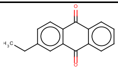	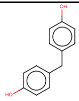	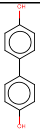		
	LD50	1000	2795	4950	4920		
	LOEL	125	10	250	40	100.00	1.25
20	Structure	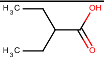	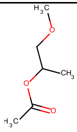	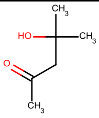	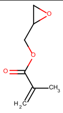		
	LD50	2200	8532	2520	500		
	LOEL	50	1000	100	30	376.67	7.53

Table S6. *Cont.*

Sr	Data	Target	Ana-1	Ana-2	Ana-3	LOEL_pred	Fold_diff
21	Structure	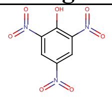	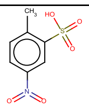	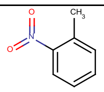	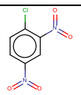		
	LD50	200	3710	891	640		
	LOEL	100	350	625	6	327.00	3.27
22	Structure	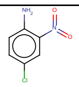	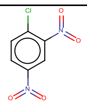	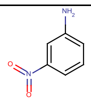	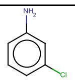		
	LD50	400	640	535	256		
	LOEL	10	6	15	10	10.33	1.03
23	Structure	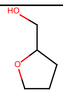	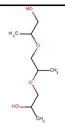	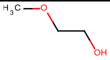	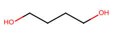		
	LD50	1600	3000	2370	1525		
	LOEL	150	1000	750	200	650.00	4.33
24	Structure	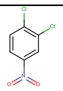	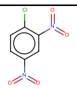	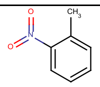	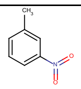		
	LD50	953	640	891	1072		
	LOEL	4	6	625	625	418.67	104.67
